# A ten-gene signature-based risk assessment model predicts the prognosis of lung adenocarcinoma

**DOI:** 10.1186/s12885-020-07235-z

**Published:** 2020-08-20

**Authors:** Hanliang Jiang, Shan Xu, Chunhua Chen

**Affiliations:** Department of Pulmonary and Critical Care Medicine, Sir Run Run Shaw Hospital, Zhejiang University School of Medicine, No. 3 Eastern Qingchun Road, Hangzhou, 310016 China

**Keywords:** LUAD, Feature gene, Risk assessment model, Prognosis prediction

## Abstract

**Background:**

Lung adenocarcinoma (LUAD) is a major cause of cancer death. Therefore, identifying potential prognostic risk factors is critical to improve the survival of patients with LUAD.

**Methods:**

Here, relevant datasets were downloaded from TCGA and GEO databases to screen the differentially expressed genes (DEGs). Univariate Cox analysis, LASSO regression analysis and multivariate Cox analysis were conducted on the DEGs combined with TCGA clinical data, and finally a risk assessment model based on 10 feature genes was constructed.

**Results:**

The prognosis of patients was evaluated after the patients were grouped based on the median risk score and the results showed that the survival time of patients in the high-risk group was significantly shorter than that in the low-risk group. ROC analysis showed that the AUC values of the 1, 3, 5-year survival were 0.753, 0.724, and 0.73, respectively, indicating that the model was precise in predicting the prognosis, which was also verified in the external dataset GSE72094. In addition, a significant correlation was found between the risk score and the clinical stages of LUAD, that is, a later stage always corresponded to a higher risk score. Then, we performed survival analysis on the 10 feature genes independently in the TCGA-LUAD dataset through the GEPIA database, finding that the high expression of 6 genes (COL5A2, PLEK2, BAIAP2L2, S100P, ZIC2, SFXN1) was associated with the poor prognosis of LUAD patients.

**Conclusion:**

To sum, this study established a 10-gene risk assessment model and further evaluated its value in predicting LUAD prognosis, which provided a new method for the prognosis prediction of LUAD.

## Background

Lung cancer had become the most frequently diagnosed cancers worldwide, according to the latest cancer statistics released in 2018 [1]. Non-small cell lung cancer (NSCLC) and small cell lung cancer (SCLC) are two subtypes of lung cancer. Lung adenocarcinoma (LUAD) and lung squamous cell carcinoma (LUSC) are the two main types of NSCLC [2], while LUAD accounts for a higher proportion [3]. With the development of molecular targeted therapy and immunotherapy, the survival rate of LUAD has been gradually improved. For example, tyrosine kinase inhibitors (TKIs) targeting epidermal growth factor receptor (EGFR) have been considered as the standard first-line treatment of advanced LUAD in patients with sensitive EGFR gene mutations [4]. ROS proto-oncogene 1 (ROS1) and anaplastic lymphoma kinase (ALK) gene are common oncogenes in the targeted therapy of LUAD [5]. In addition, approved immunotherapy for lung cancer is aimed at the reversal of immune checkpoints, programmed death protein-1 (PD-1) and programmed death ligand-1 (PD-L1), and it has a good therapeutic effect in specific lung cancer patients [6]. However, despite the continuous improvement in LUAD treatment, the 5-year overall survival (OS) rate is still at a low level with unoptimistic prognosis [7, 8]. In clinical practice, histopathology is often successful in predicting the prognosis of lung cancer patients, but it is limited as individual differences in patients with the same pathology would cause different outcomes. Combined with existing prognostic methods, new molecular biomarkers are considered to have the capability of improving prognosis and treating LUAD appropriately. Therefore, screening more molecular biomarkers is of great importance.

In recent years, more and more prognostic biomarkers for LUAD have been found by analyzing the clinical information and expression profiles in public databases [9–11]. Wei et al. identified 151 differentially methylated genes related to relapse-free survival of patients with LUAD by analyzing TCGA expression profiles and nine hub genes were identified in the PPI network, among which a 4-gene pair signature was identified as a prognostic biomarker for patients with stage I LUAD [12]. Chang et al. identified four glycolytic genes (AGRN, AKR1A1, DDIT4 and HMMR) that are closely related to the prognosis of LUAD patients by analyzing the expression profiles of LUAD patients in TCGA database [13]. In addition, Fuduan et al. developed a prognostic signature consisting of two lncRNAs (C1orf132 and TMPO-AS1) for stage I-II LUAD patients without receiving adjuvant therapy, which was further confirmed in two independent datasets of GSE50081 and GSE31210 [14]. These studies indicate that using public database sources to develop prognostic risk models has a great potential. However, the effectiveness of these diagnostic models for clinical practice has not been tested. Thus, it is necessary to continue to mine genes and polygenic signatures associated with LUAD prognosis.

In this study, we downloaded the LUAD-related mRNA expression profiles from TCGA database and a relevant GEO dataset to screen the differentially expressed genes (DEGs). Univariate Cox combined with LASSO regression analyses were used to screen out feature genes related to the prognosis of LUAD patients, and multivariate Cox models were established to build an optimal 10-gene signature-based risk assessment model to evaluate the survival of LUAD patients. Our study provides a new method to assist the prediction of prognosis in clinical LUAD patients.

## Methods

### DEGs screening

mRNA expression profiles (including 535 tumor samples and 59 normal samples) and clinical data (the download time was 9th December, 2019) of LUAD were downloaded from TCGA database (http://ualcan.path.uab.edu/cgi-bin/ualcan-res.pl). R-package “edgeR” was used to screen the DEGs based on the mRNA expression profiles and the normal samples were set as the control (|logFC| > 1.5, padj< 0.05). Meanwhile, GSE75037 (including 83 tumor samples and 83 non-tumor samples), a LUAD-related dataset, was downloaded from GEO database (https://www.ncbi.nlm.nih.gov/geo/), and the R-package “limma” was used to screen the DEGs with the threshold of |logFC| > 1.5 and padj< 0.05. During the process of model establishment, the tumor samples with incomplete survival time or state were removed. While in the correlation analysis with clinicopathologic characteristics of LUAD patients, “unknown”, “TX”, “NX” and other samples were removed.

### GO and KEGG enrichment analyses

GO and KEGG functional enrichment analyses were performed on the DEGs using the DAVID 6.8 software, and the pathways with a *P* value less than 0.05 were selected as the most enriched GO and KEGG pathways significantly related to biological functions of LUAD cells.

### Univariate cox and LASSO regression analyses

Combined with the clinical information of LUAD in TCGA database, the genes related to the prognosis of LUAD patients were screened from the obtained DEGs. In other words, all the DEGs were analyzed by univariate Cox regression analysis and *p* < 0.01 was used as cutoff to screen out the prognosis-related genes. In order to prevent the phenomenon of over-fitting in the modeling process of multivariate Cox regression models, LASSO regression analysis was conducted on the prognosis-related genes, and the penalty parameter “lambda” was selected by cross validation method.

### Risk assessment model construction and evaluation

The R-package “Survival” was used to construct multiple multivariate Cox models based on the feature genes selected by LASSO regression analysis, and the optimal risk assessment model composed of 10 genes were identified. According to the risk model, samples in the TCGA were given a score and then divided into high-risk group and low-risk group with the median risk score as threshold. The survival curves of the patients in the high and low risk groups were drawn with the R-package “Survival”, and the survival time of the two groups was compared by log-rank test. ROC curves were drawn using the R package “survivalROC” for validation of the risk model and the AUC values of 1, 3 and 5-year survival were calculated. Furthermore, survival analysis was conducted on the 10 individual feature genes in the TCGA-LUAD dataset using the GEPIA database. Two independent datasets GSE72094 and GSE31210 were used for further validation of the 10-gene risk model.

## Results

### Identification of DEGs and GO and KEGG pathway enrichment analyses

**Fig. 1:**
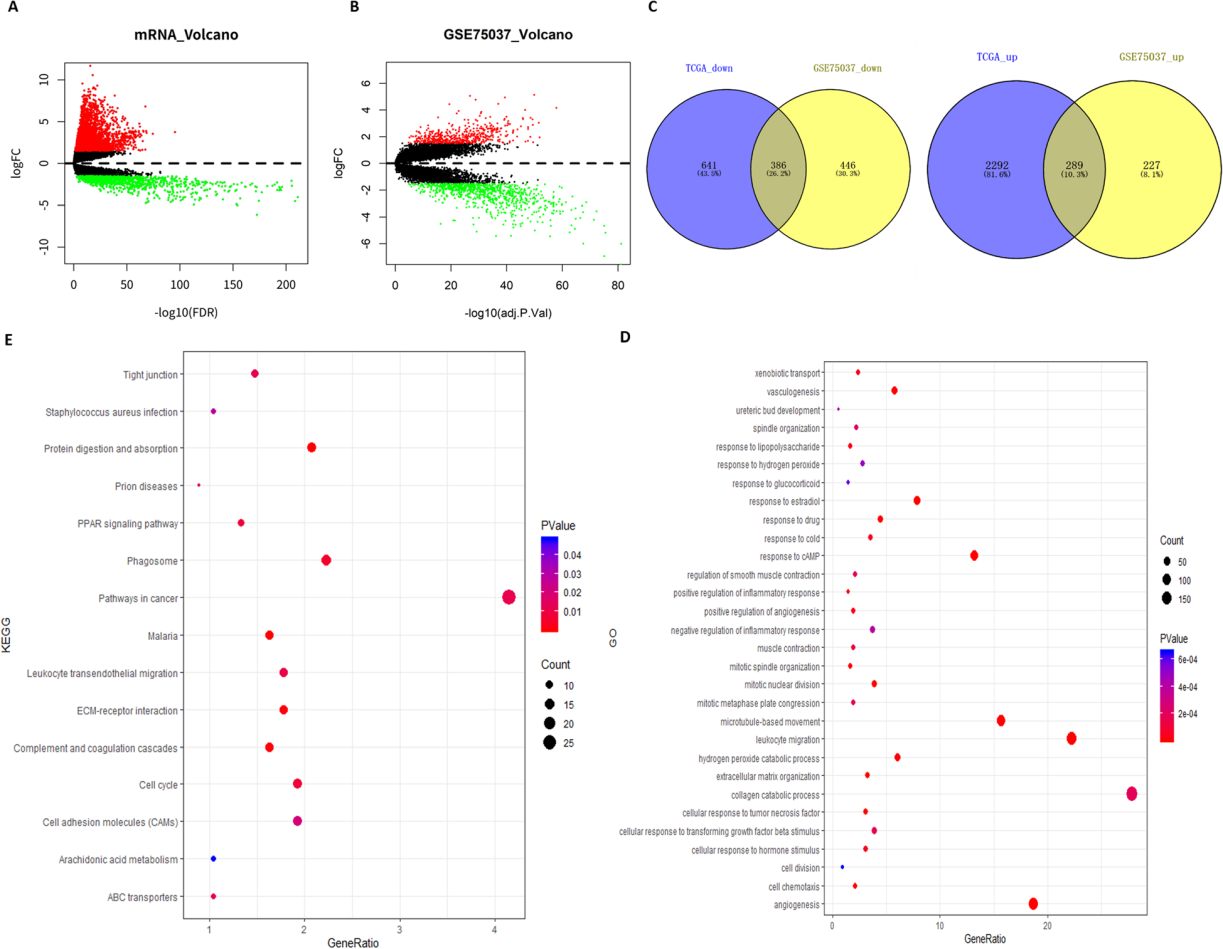
GO and KEGG pathway enrichment analyses are carried out on the screened DEGs. The volcano plots of DEGs obtained in TCGA database **a** and dataset GSE75037 **b** (Red dots represent up-regulated genes and green dots represent down-regulated genes); The Venn diagram **c** of the DEGs in TCGA database and dataset GSE75037; GO **d** and KEGG **e** pathway enrichment analyses results of the overlapping DEGs

mRNA expression profiles and clinical data of LUAD were downloaded from TCGA database, and eventually 3608 DEGs were obtained by differential analysis using R-package (Fig. 1a). Meanwhile, 1348 DEGs were obtained from the dataset GSE75037 (Fig. 1b). From the intersection of the two datasets, a total of 675 DEGs were overlapped, including 386 downregulated genes and 289 upregulated genes (Fig. 1c).

In order to analyze the functions regulated by the DEGs in LUAD patients from the level of biological functions, GO and KEGG functional enrichment analyses were performed on the 675 DEGs. Identifying the biological functions of these DEGs is of great significance to analyze the pathogenesis of LUAD. GO enrichment analysis result showed that the DEGs were mainly enriched in cell division, mitosis, angiogenesis and other biological functions associated with cell proliferation and invasion (Fig. 1d). KEGG enrichment analysis result indicated that the DEGs were mainly enriched in cell cycle, ECM receptor interactions, cell adhesion molecules and other biological functions related to cell proliferation and invasion (Fig. 1e). These suggested that the DEGs were most likely associated with tumor proliferation and metastasis.

### Prognosis-related genes are screened to construct a 10-gene risk assessment model for predicting the prognosis of LUAD

**Fig. 2:**
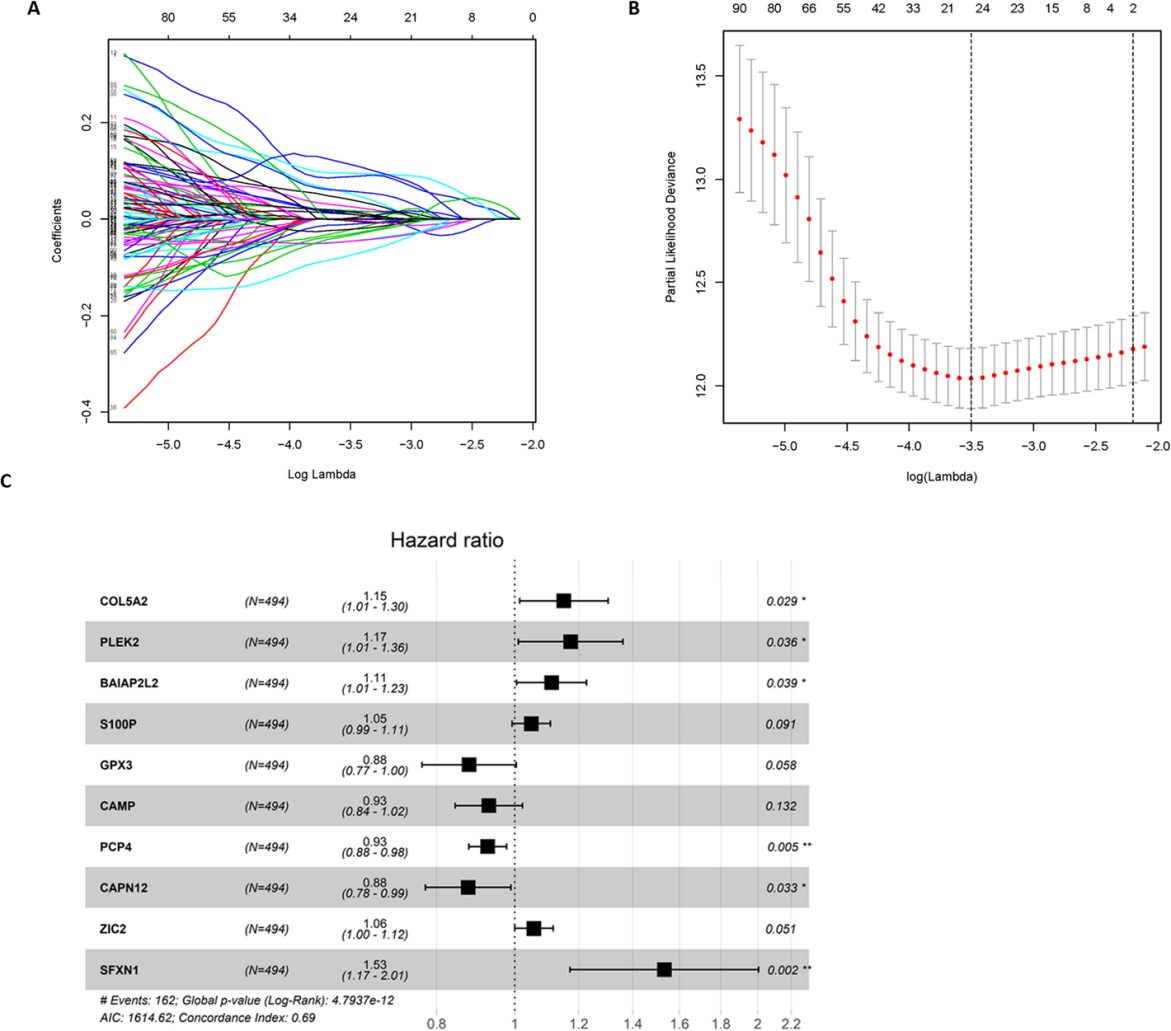
Prognosis-related genes are screened and a risk assessment model is constructed. **a** The LASSO regression model shows the genes associated with LUAD survival when log lambia approaches 0; **b** The penalty coefficient interval is used to minimize the mean square error of the model; **c** The forest map of the multivariate Cox analysis on the 10 independent prognostic feature genes

Combined with the clinical information of LUAD in TCGA database, genes related to the prognosis of LUAD patients were screened from the 675 DEGs. One hundred forty-four genes were screened by univariate Cox analysis and *P* < 0.01 was used as cutoff (**Supplementary Table** **1****)**. LASSO Cox regression analysis was performed on the 144 DEGs, and the penalty parameter lambda was selected by cross validation method to obtain 24 relatively independent feature genes for subsequent model analysis (Fig. 2a, b). The result of LASSO regression analysis was exhibited in **Supplementary Table** **2**.

Multivariate Cox regression models were established based on the 24 feature genes using the R-package “Survival”, and finally 10 genes (COL5A2, PLEK2, BAIAP2L2, S100P, GPX3, CAMP, PCP4, CAPN12, ZIC2, SFXN1) were selected as independent prognostic factors for LUAD (Fig. 2c). Six significant digits were reserved for the coefficients in the model, and the product of gene expression and corresponding coefficient of each gene was added to establish a risk score: riskscore = 0.140049*EXP (COL5A2) + PLEK2*EXP (GPR37) + (− 0.0964595) *EXP (BAIAP2L2) + (− 0.115410) *EXP (S100P) + 0.0886797*EXP (GPX3) + (− 0.070677) *EXP (CAMP) + 0.144484*EXP (PCP4) + 0.158681*EXP (CAPN12) + 0.0731869*EXP (ZIC2) + 0.0614746*EXP (SFXN1). Multivariate Cox results were listed in **Supplementary Table** **3**. The risk score of each sample was calculated based on these 10 independent prognostic feature genes.

### The predictive ability of the 10-gene risk assessment model is evaluated

**Fig. 3:**
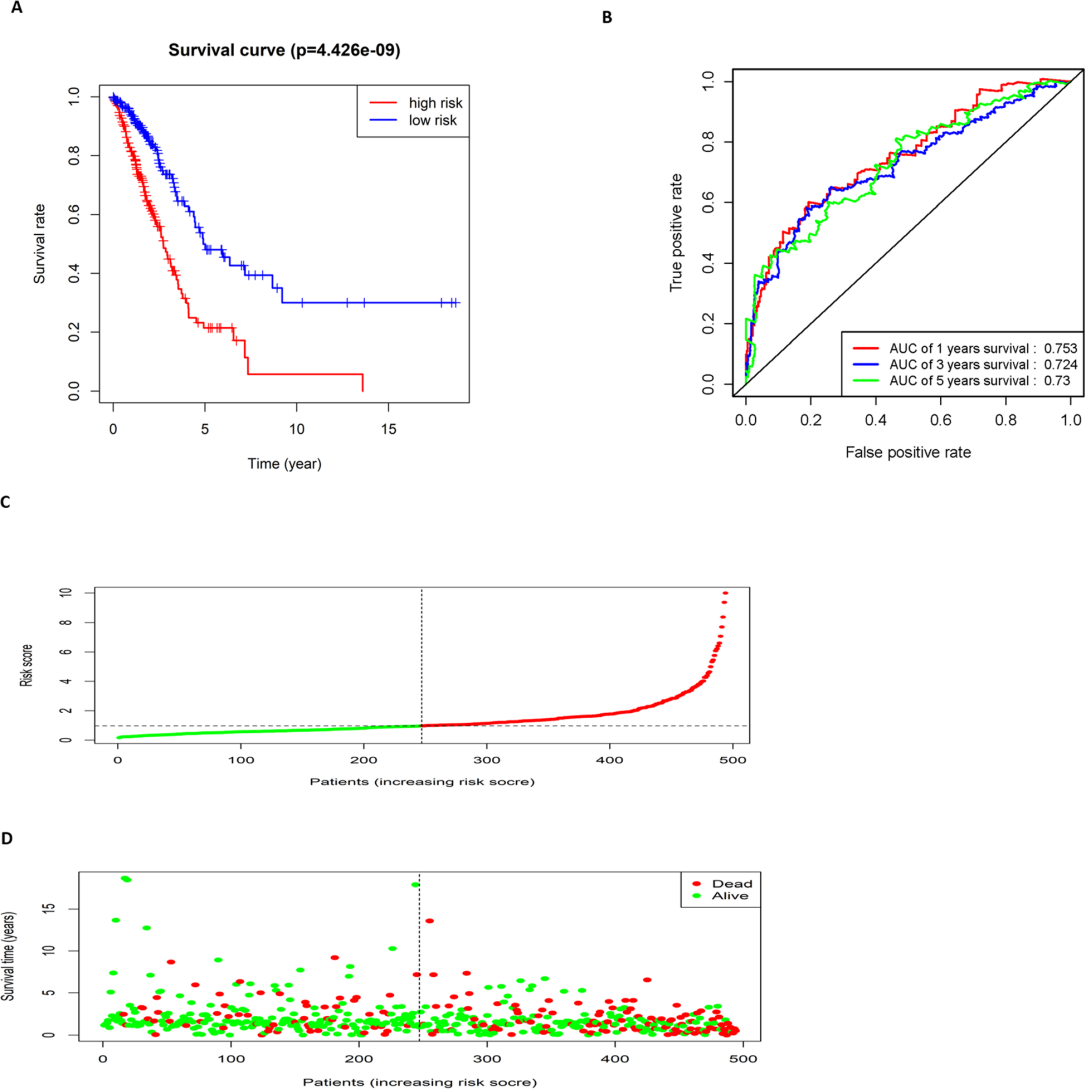
The risk assessment model predicts the survival time and survival status of LUAD patients. **a** Kaplan-Meier survival curves of the patients with a high risk score (red) and a low risk score (blue); **b** ROC curves show the 1-year (red), 3-year (blue), and 5-year (green) survival of LUAD patients using the 10-gene risk score model; **c** The risk score distribution of each LUAD sample (The green dots represent patients with a low risk score and the red dots represent patients with a high risk score); **d** The scatter diagram shows the survival of LUAD patients according to the risk score (The green dots represent survived patients and the red dots represent deaths)

The samples were divided into the high-risk group and low-risk group according to the median risk score, and the survival curves of the two groups were drawn to compare the survival time. The result exhibited that the survival time of the high-risk group was significantly shorter than that of the low-risk group (Fig. 3a).

Then, ROC curves were drawn to verify the risk assessment model, and the AUC values of 1, 3 and 5-year survival were 0.753, 0.724 and 0.73, respectively (Fig. 3b). It was proved that the risk model based on these 10 feature genes could predict the prognosis of LUAD patients. The risk score distribution of each sample was shown in Fig. 3c. We also drew a scatter diagram showing the survival time of patients based on the risk score, and found that with the increase of the risk score, the number of death increased and the survival time of patients also gradually decreased (Fig. 3d). The above results suggested that the 10-gene signature-based risk assessment model had certain predictive value for the prognosis of LUAD patients, and a higher risk score resulted in a worse prognosis.

### Correlation analysis between the risk assessment score and clinicopathologic features of LUAD patients

**Fig. 4:**
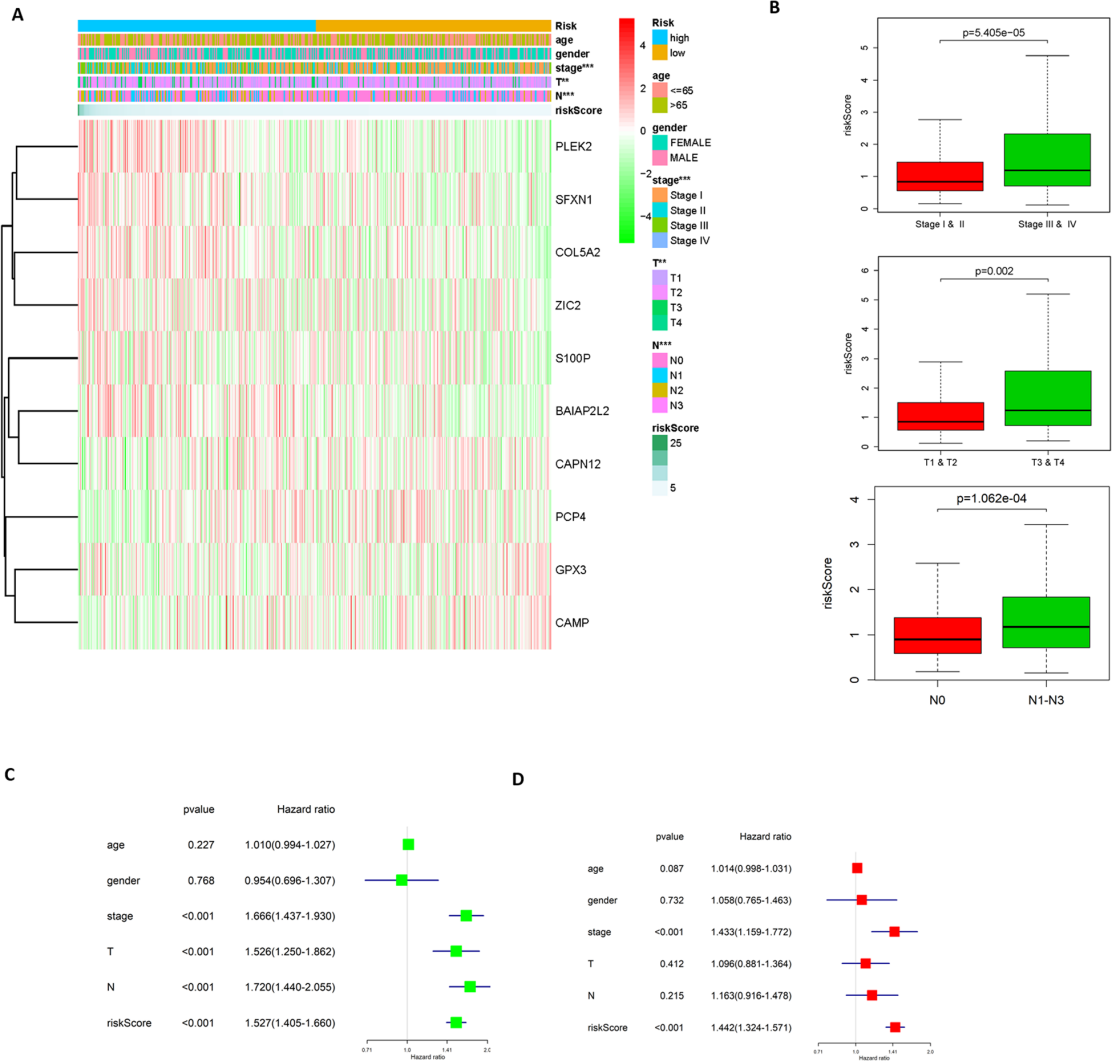
Correlation analysis between the risk assessment score and clinicopathologic features of LUAD patients. **a** The expression heat map of the 10 feature genes in the high and low risk groups and the clinicopathologic differences between the two groups; **b** Boxplots show the risk assessment score of patients with different pathological stage, T_stage and N_ stage; The forest maps of the **c** univariate and **d** multivariate regression analyses on the 10-gene risk score combined with clinical information

The expression heat map of the 10 feature genes in the high and low risk groups was plotted and the clinicopathologic differences between the two groups were shown in the heat map as well. The results concluded that with the increase of the risk score, the expression levels of PLEK2, SFXN1, COL5A2, ZIC2, SL100P and BAIAP2L2 gradually increased, while the expression levels of CAPN12, PCP4, GPX3 and CAMP gradually decreased. Moreover, there were significant differences between the high-risk group and the low-risk group in different pathological stage, T_stage and N_ stage (Fig. 4a). A higher tumor stage was accompanied by a higher risk score (Fig. 4b). The above findings further demonstrated that the 10-gene model could predict the risk of LUAD.

Univariate analysis was conducted based on the risk score of the 10-gene model and clinical information. The result displayed that risk score, pathological stage, T_stage and N_ stage had significant effects on prognosis (Fig. 4c). While the result of multivariate analysis demonstrated that only risk score and pathological stage had significant significance for prognosis (Fig. 4d). Taken together, it indicated that the 10 -gene signature-based model was closely related to tumor stages and could be used as an independent prognostic factor for LUAD patients.

### Survival analysis of the 10 feature genes in the model

**Fig. 5:**
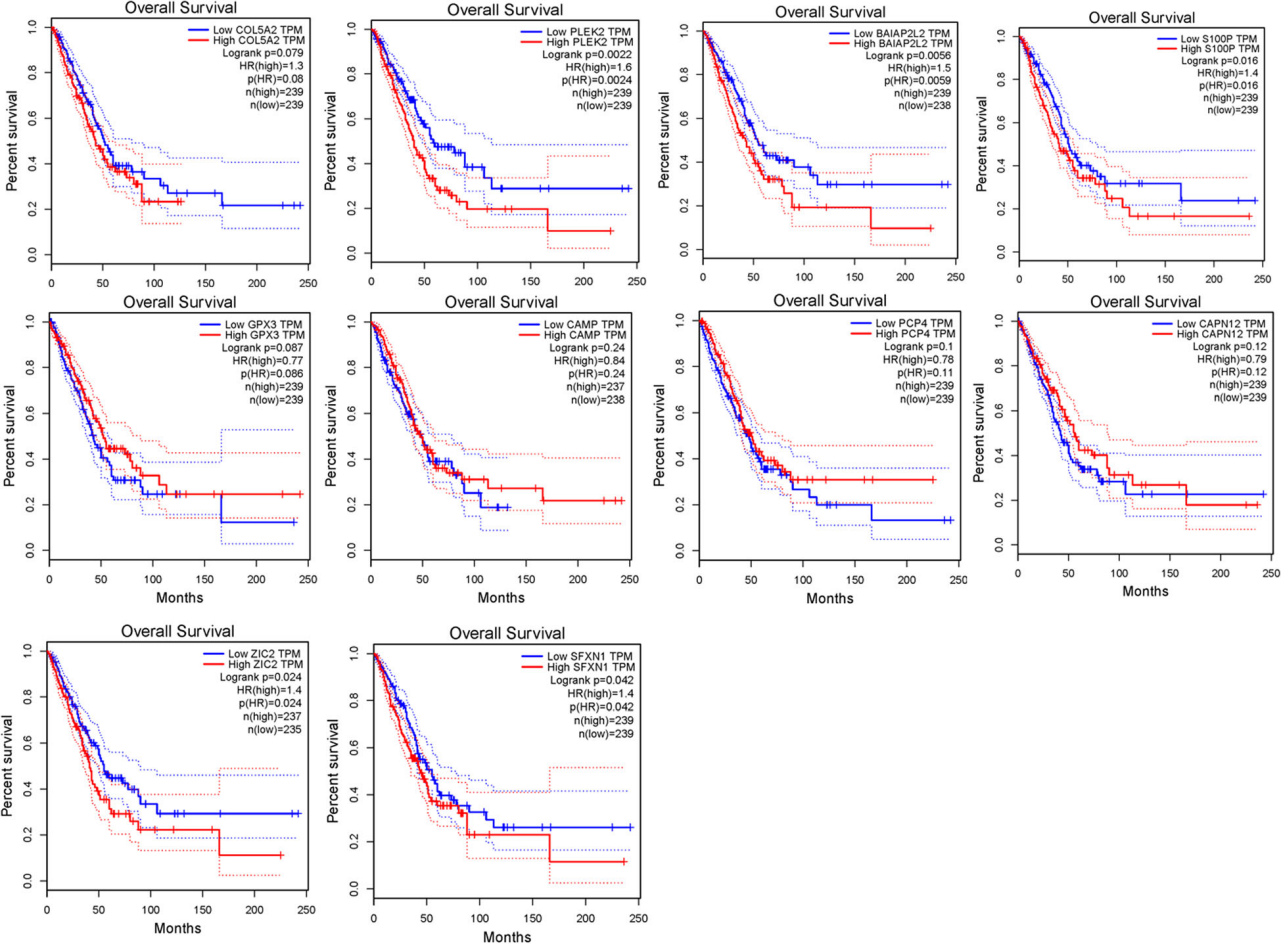
Kaplan-Meier survival analysis is performed on the 10 individual genes

To verify the significance of the expression of the 10 feature genes in predicting the prognosis of LUAD, the GEPIA database was used to conduct survival analysis on the 10 feature genes in the TCGA-LUAD dataset. The results proved that except the four low-risk factors in the model (GPX3, CAMP, PCP4, CAPN12), the expression of the other six high-risk genes were significantly negatively correlated with the prognosis (Fig. 5). This may indicate that the expression of the six high-risk genes in this model had a greater effect on prognosis.

### Dataset GSE72094 and GSE31210 are used to validate the 10-gene model

**Fig. 6:**
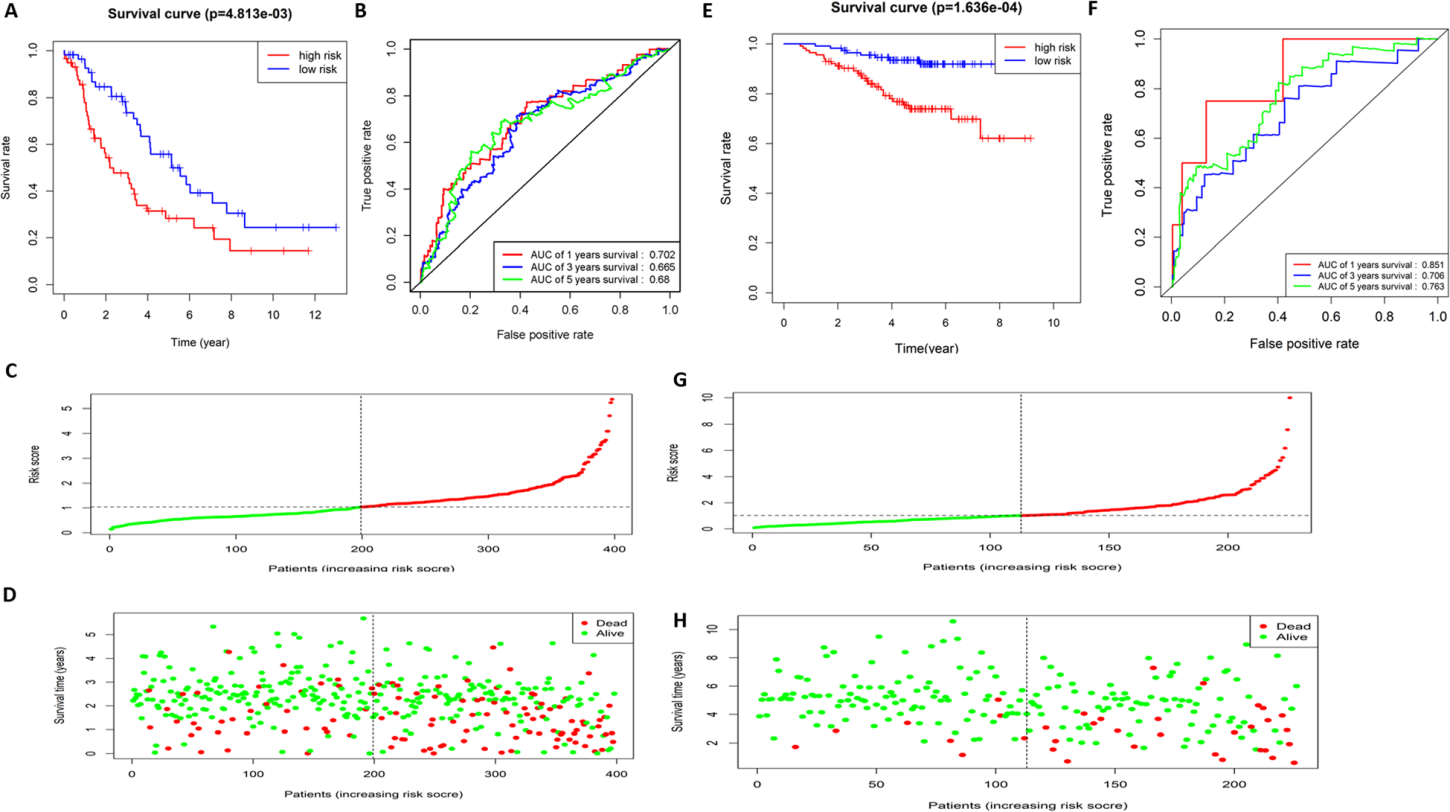
The risk assessment model is validated using two independent datasets GSE72094 and GSE31210. Kaplan-Meier survival curves show the effect of the 10-gene risk score on the survival time of LUAD patients in GSE72094 **a** and GSE31210 **e** datasets (Red represents the patients with a high risk score and blue represents the patients with a low risk score); ROC curves showing the 1-year (red), 3-year (blue), and 5-year (green) survival of LUAD patients were plotted using the 10-gene risk score in GSE72094 **b** and GSE31210 **f** datasets; The risk score distribution of each LUAD sample in GSE72094 **c** and GSE31210 **g** datasets (The green dots represent the patients with a low risk score and the red dots represent the patients with a high risk score); The scatter diagram shows the survival of LUAD patients with different risk scores in GSE72094 **d** and GSE31210 **h** datasets, with green dots representing survived patients and red dots representing deaths

Based on the 10-gene signature-based model, patients in GSE72094 (including 442 patients with LUAD) and GSE31210 (including 226 patients with LUAD) datasets were given a score using multivariate Cox regression analysis. The samples were divided into the high-risk and low-risk groups according to the median risk score, and survival curves of the two groups were drawn to compare the survival time. The results showed that the survival time of the patients in the high-risk group was significantly shorter than that of the patients in the low-risk group in both two datasets (Fig. 6a, e).

ROC curves were drawn to verify the model reliability, and the AUC values for 1, 3 and 5-year survival were 0.702, 0.665, 0.68 (GSE72094) and 0.851, 0.706, 0.763 (GSE31210), respectively (Fig. 6b, f). It indicated that the 10-gene risk assessment model had a good predictive ability for the prognosis of LUAD patients in the two independent datasets GSE72094 and GSE31210. The risk score distribution of the samples in the GSE72094 and GSE31210 datasets were exhibited in Fig. 6c and g. We further plotted a scatter diagram showing the survival of patients with different risk scores, and the result showed that with the increase of the score, the number of death event increased gradually, which was supported by the previous research (Fig. 6d, h).

## Discussion

**Fig. 7:**
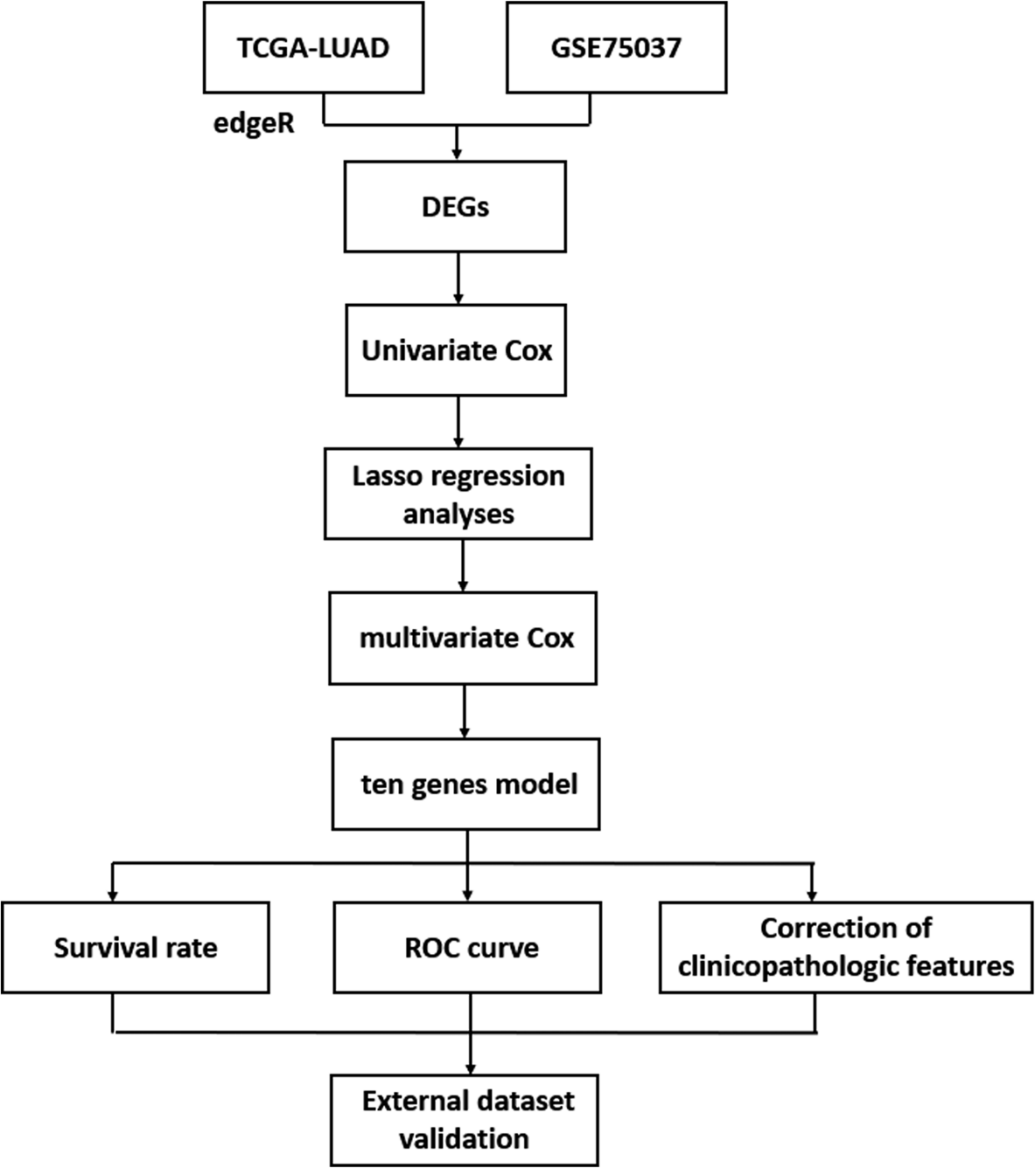
A risk assessment model composed of 10 feature genes

Most lung cancer patients are diagnosed at an advanced stage, while metastasis and drug resistance always appear in the early stages of treatment [15, 16]. Different lung cancer subtypes present different clinical characteristics and prognosis, thus, it is vital to explore prognostic markers specific to LUAD. In this study, through a series of analyses on the DEGs associated with LUAD, we finally developed a risk assessment model composed of 10 feature genes **(**Fig. 7**)**, and the risk score was formulated as shown in the section of 2.2. To further verify the reliability of the model, we divided the samples into the high-risk group and low-risk group according to the median risk score, and studied the prognosis of patients in the two groups. The results demonstrated that the survival time of patients in the high-risk group was significantly shorter than that in the low-risk group. ROC curves were used to evaluate the performance on predicting prognosis and the result showed that the AUC values of the 1, 3, 5-year survival were 0.753, 0.724, and 0.73, respectively, indicating that the model was of good accuracy, which was also verified in the two independent datasets GSE72094 (1-year AUC = 0.702, 3-year AUC = 0.665, 5-year AUC = 0.68) and GSE31210 (1-year AUC = 0.851, 3-year AUC = 0.706, 5-year AUC = 0.763). Subsequently, the correlation between the risk score and clinicopathologic characteristics was investigated, and it was found that a later stage of LUAD was accompanied by a higher risk score, which further demonstrated the predictive potential of the risk assessment model.

All the 10 genes in the model were DEGs in LUAD, and the DEGs in LUAD were mainly enriched in the signaling pathways closely related to cell proliferation, invasion and migration. Therefore, we speculated that these 10 genes might be related to the development and prognosis of LUAD. We conducted survival analysis on these 10 genes, and found that 6 of them (COL5A2, PLEK2, BAIAP2L2, S100P, ZIC2, SFXN1) were significantly correlated with the prognosis of LUAD patients, and patients with the high expression of these 6 genes were often accompanied by a poor prognosis. Therefore, these 6 genes were emphatically concerned.

Existing studies have reported that most of these 6 key genes are closely related to the development of multiple cancers. COL5A2 has different roles in predicting the prognosis of different cancers. A retrospective analysis of the gene expression profiles related to bladder cancer shows that COL5A2 is associated with the poor clinical prognosis and a low survival rate of patients with bladder cancer [17]. Reversely, COL5A2 may be a favorable factor for the prognosis of tongue squamous cell carcinoma [18]. PLEK2 redistributes actin in cells and induces cell diffusion [19]. In addition, it is also closely associated with cancer invasion and migration [20]. Besides, PLEK2 mediates metastasis and vascular invasion via the ubiquitin-dependent degradation of SHIP2 in NSCLC [21]. S100P is related to the proliferation and migration of nasopharyngeal carcinoma cells. Additionally, reduced S100P expression induces the down-regulation of epidermal growth factor receptor, cluster of differentiation (CD) 44, matrix metalloproteinase (MMP) 2 and MMP9 protein expression [22]. Other studies found that the expression of S100P in LUAD is up-regulated, and the interaction between extracellular S100P and receptor for activated glycation end products (RAGE) contributes to tumor development [23]. Moreover, S100P can also be used as a prognostic marker for breast cancer [24]. ZIC2 can promote the malignant progression of various cancers, such as liver cancer [25, 26], nasopharyngeal cancer [27], breast cancer [28], cervical cancer [29] and so on. SFXN1 is a mitochondrial serine transporter required for carbon metabolism [30]. It is unknown whether SFXN1 and BAIAP2L2 are involved in the cancer process as few studies on these two genes have been reported. In view of the important role of these feature genes in cancer, we can further study the specific mechanisms of them in LUAD in the future.

## Conclusion

This study established a 10-gene risk assessment model and evaluated its good performance on predicting the prognosis of LUAD. The multi-gene signature-based risk assessment model is more accurate than the single-gene prognostic marker, and the model built in this study provides a new method for evaluating the survival and prognosis of patients with LUAD. However, due to the epidemiological limitations, we were unable to detect the specific association between the simulated risk score and the prognosis of LUAD patients, and have not yet been clinically verified it. Therefore, the verification of the 10-gene risk assessment model and the research on the regulatory mechanism of single genes in this model need to be further carried out. In conclusion, our study provides a new auxiliary method for predicting the prognosis and a new direction for exploring therapeutic targets of LUAD.

## Supplementary information

**Additional file 1.**

**Additional file 2.**

**Additional file 3.**

## Data Availability

The data used to support the findings of this study are included within the article. The data and materials in the current study are available from the corresponding author on reasonable request.

## Abbreviations

LUAD: Lung adenocarcinoma
DEGs: Differentially expressed genes
NSCLC: Non-small cell lung cancer
SCLC: Small cell lung cancer
LUSC: Lung squamous cell carcinoma
TKIs: Tyrosine kinase inhibitors
EGFR: Epidermal growth factor receptor
ALK: Anaplastic lymphoma kinase
OS: Overall survival
